# Short-term clinical and radiological results of two different design metaphyseal fitting femoral stems in total hip arthroplasty: a prospective, randomized trial

**DOI:** 10.1186/s13018-021-02465-8

**Published:** 2021-05-17

**Authors:** I. Tatani, K. Solou, A. Panagopoulos, J. Lakoumentas, A. Kouzelis, P. Megas

**Affiliations:** 1 Orthopaedic Department, University Hospital of Patras, Papanikolaou 1, Rio-Patra, 26504 Patras, Greece; 2 Laboratory Department of Medical Physics, School of Medicine, University of Patras, Patras, Greece

**Keywords:** Hip replacement, Short stem, Clinical outcome, Radiological analysis, Metaphyseal femoral implants, Prospective

## Abstract

**Background:**

There is great design variability on short femoral stems available on the market. This study aims to evaluate the short-term clinical and radiological results of two different design short femoral stems, both classified as shortened tapered stems.

**Methods:**

From March 2016 to March 2018, a prospective, randomized, parallel-group design study was conducted to compare functional and radiological outcomes of 45 patients underwent THA using the Tri-Lock Bone Preservation Stem and 45 patients underwent THA with the Minima S stem at a minimum 2 years of follow-up. Patients were assessed clinically and radiographically prior to surgery as well as at 3, 6, 12, and 24 months postoperatively. Primary outcomes were the change in health-related quality of life assessed with Western Ontario and McMaster Universities Osteoarthritis Index and 36-Item Short Form Health Survey and also the incidence of all hip-related complications. Secondary aims included hip function evaluated with the Harris hip score, rates of patient satisfaction, and the outcomes of a detailed radiological analysis.

**Results:**

There were no significant differences between the 2 study groups in terms of patient-reported outcomes measurements, satisfaction scores, and complication rates at any of the measurement times. In both groups, stable fixation and radiographic osseointegration were achieved. However, analysis of the calcar region showed that 57.8% and 28.9% of patients had grade 1 or 2 stress shielding, in Tri-Lock and Minima S implantation group, respectively (*p*=0.015). Regarding coronal alignment, stems were placed in slight varus, valgus, and neutral position in 51.1%, 13.3%, and 35.6% of patients, respectively, in Tri-Lock BPS group. The Minima S stem was implanted at slight varus and valgus in 60% and 40% of patients, respectively, and neither stem in the exact neutral position.

**Conclusions:**

Both different design short femoral stems demonstrated excellent clinical performance at short-term follow up. Nevertheless, concerns were raised regarding the incidence of stress shielding phenomenon and mild discrepancies in coronal stem alignment during implantation. The clinical impact of these observations should be further evaluated through larger cohorts and longer follow-up.

**Trial registration:**

ISRCTN registry, ISRCTN10096716. Registered on May 8, 2018—Retrospectively registered

## Background

Modern total hip arthroplasty (THA) has been shown to generate good long-term clinical outcomes and survival rates and undoubtedly has been considered the operation of the century. Advances in materials, implant designs, and surgical techniques are among the factors that have enabled this success. Supporting this concept, several short femoral stems have been designed with the theoretical advantages of preserving bone stock, optimizing stress distribution at the proximal femur to reduce shielding-induced bone loss, facilitating tissue-sparing approaches, and simplifying revision procedures [1–3]. All these benefits, even theoretical, are of particular importance considering the recent shift towards younger and more active patients undergoing THA [4]. This group of patients is at higher risk for a revision procedure during lifetime, and thus, the preservation of bone tissue should be the fundamental goal of the primary operation.

In recent years, a heterogeneous group of short stems deeply different in terms of design, biomechanics, and principles of fixation has been launched in the market. In this respect, several proposals to categorize these protheses have been introduced in the literature leading to a great inhomogeneity regarding the nomenclature of these implants. In the classification system proposed by Khanuja et al [5], short stems are classified into four categories: femoral neck fixation, calcar loading, lateral flare and calcar loading, and shortened tapered stems, according to the location of proximal loading and the principles of fixation of each prosthesis. However, short stems present differences in terms of design, fixation, biomechanics, and clinical outcomes, even if they belong to the same main category [5–8]. To date, only short to mid-term clinical results are available for certain designs of short stems.

This study aims to compare the functional and radiological results of short stem THA using two metaphyseal fitting short stems, type 4 according to the classification system proposed by Khanuja et al. [5], the Tri-Lock Bone Preservation Stem (DePuy Orthopaedics Inc. Warsaw, IN, USA) and the Minima S Femoral Stem (Lima corporate Villanova di San Daniele, Italy). Primary goals were to evaluate the incidence of all hip-related complications and change in health-related quality of life assessed with Western Ontario and McMaster Universities Osteoarthritis Index (WOMAC) [9] and 36-Item Short Form Health Survey (SF-36) [10] scores up to 2 years postoperatively. Secondary aims included hip function evaluated with the Harris hip score (HHS) [11], patient satisfaction, and a detailed radiological analysis.

## Methods

### Study design

We conducted a prospective, randomized, parallel-group designed study with blinded treatment and assessment to compare functional and radiological outcomes of two different design metaphyseal fitting short femoral stems, the Tri-Lock Bone Preservation Stem (BPS) and the Minima S Femoral Stem. The study protocol was approved by the ethics committee of the University Hospital of Patras (approval number: 36/02-03-2016), and a detailed informed consent form was signed by each patient prior to participation in the study. The approval was published in the Greek Transparency Portal, called “diavgeia” on April 22, 2016, with a unique Internet Uploading Number that was ADA:6ΝΩ346906Γ-Φ6Ω. The study was registered with the International Standard Randomized Controlled Trial Number ISRCTN10096716. The study protocol has already been peer-reviewed and published [12].

### Study population

From March 2016 to March 2018, we enrolled 143 patients aged 50–80 years old who were candidates for THA. The indication for surgery was hip osteoarthritis that was severe enough to warrant THA after an adequate trial of non-operative therapy. Patients suffering from (1) primary osteoarthritis, (2) inflammatory arthritis, (3) avascular necrosis, and (4) post-traumatic arthritis were considered eligible for inclusion. Patients were excluded in both groups if they had severe co-morbidities affecting functional outcome as well as those with poor bone stock and any femoral deformity precluding appropriate fit and fill in the metaphysis, such in cases of high-grade hip dysplasia and severe valgus or metaphyseal deformity secondary to fracture.

**Fig. 1:**
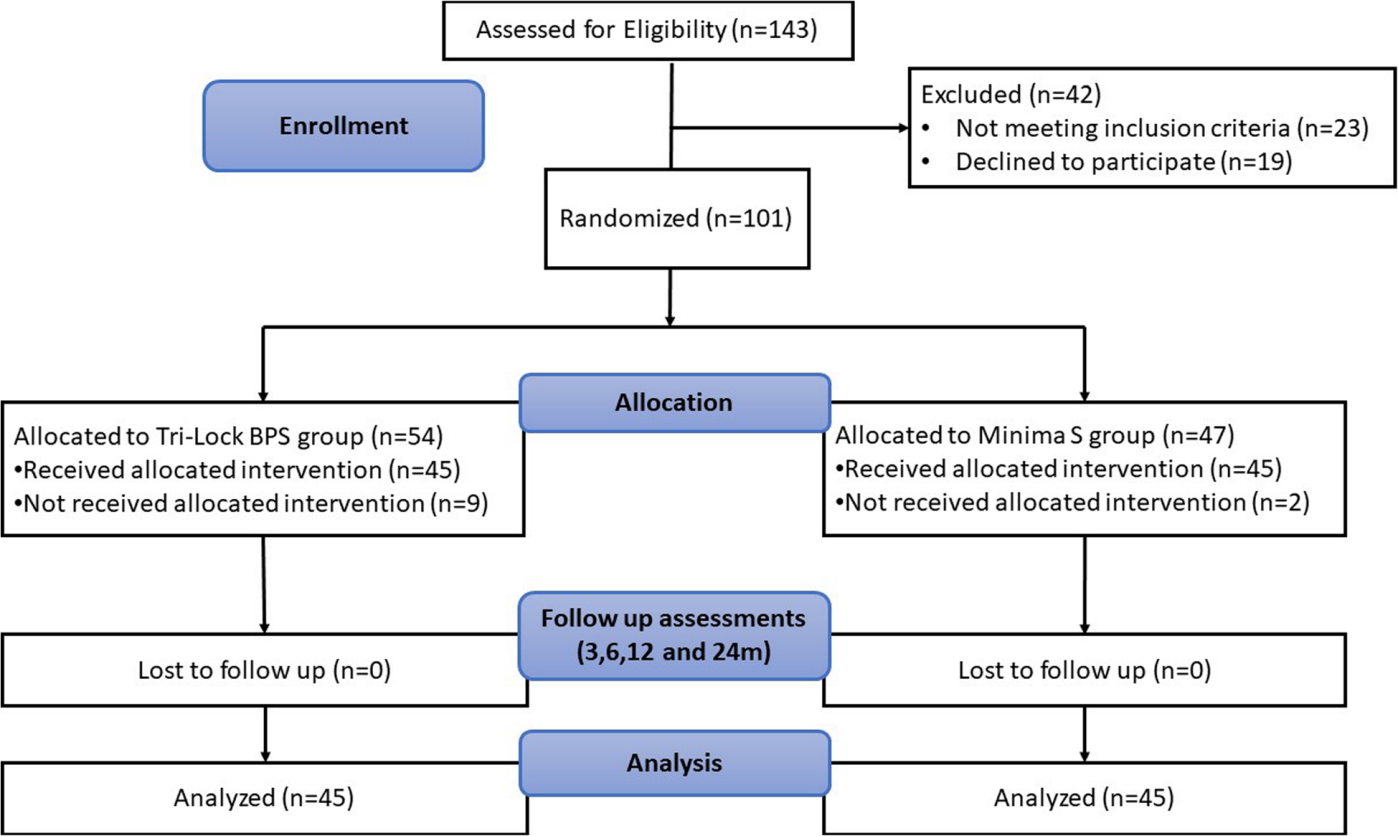
The flow diagram shows patient enrollment, allocation, follow-up, and analysis

During the study period, 101 patients were eligible and consented to enroll in this randomized clinical trial. Patients were randomly allocated, and thus, two groups of patients were created; group A: Tri-Lock BPS group and group B: Minima S Monolithic Femoral Stem group. Nine patients in the Tri-Lock BPS group and two patients in the Minima S group did not receive their allocated intervention. The remaining ninety patients were randomly allocated, and no patients were lost to follow-up across the 2-year follow-up period (Fig. 1).

### Randomization procedures

A block randomization was used, while participants were subdivided into strata, consisting of random sequence of blocks of 10 consecutive surgical procedures. Randomization was performed in the operating theatre, after anesthetic induction and just before incision, using sequentially numbered opaque sealed envelopes.

### Intervention

**Fig. 2:**
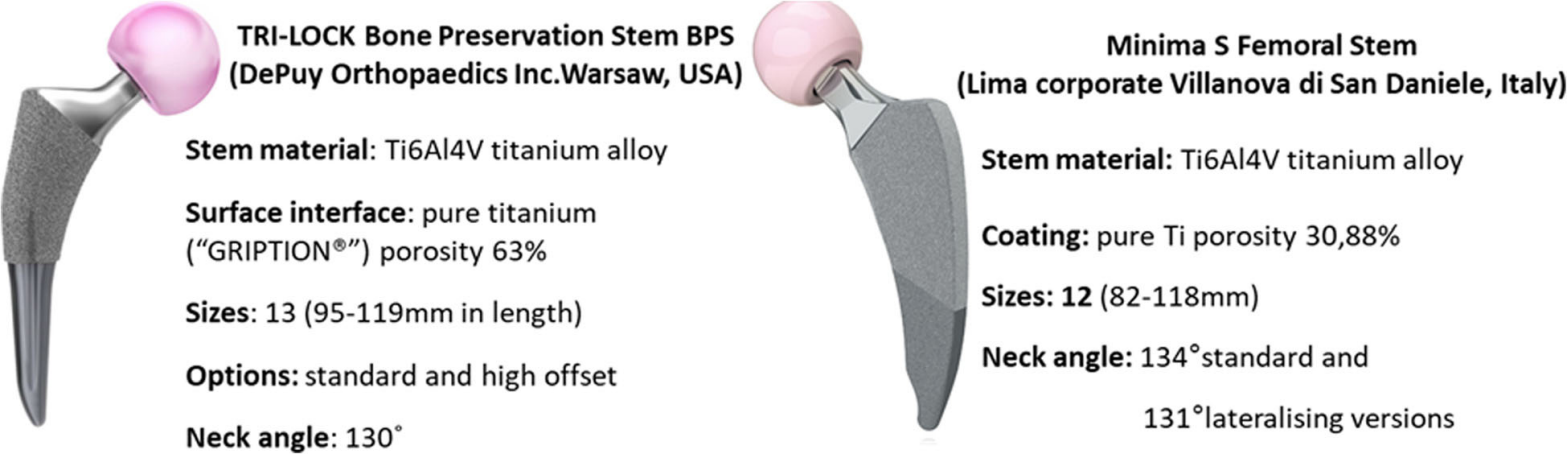
Design features of the femoral stems analyzed in the study

Preoperative templating was conducted for both implants, and both stems and their instrumentation trays were available in the operating room. The senior surgeon (P.M) performed all the arthroplasties with a standardized operative technique through a mini-posterior approach. In both groups, the femur was prepared in a broach-only fashion and then the prosthesis was impacted until a tight metaphyseal fit was obtained. The Tri-Lock BPS and Minima S femoral stems are considered shortened tapered conventional stems of type 4, according to Khanuja et al. [5] classification system. Although both stems belong to the same short stem family, some specific design differences exist (Fig. 2). The acetabulum was prepared in a standardized fashion according to the manufacturer’s instructions. In group A, all patients received a cementless Pinnacle acetabular component (DePuy, Warsaw, Indiana) while in group B, a cementless Delta PF acetabular component (Lima Corporate). All patients in both groups received a 32- or 36-mm ceramic femoral head.

All patients underwent the same postoperative physiotherapy protocol. In both groups, patients were mobilized on the second post-operative day and progressed to full-weight-bearing with a walking frame or crutches as comfort permitted; they were advised to use a walking aid for 6 weeks.

### Outcome assessment

Patients were assessed both clinically and radiographically prior to surgery. The preoperative raw data included a full demographic profile, patient age, sex, body mass index (BMI), and the Charlson’s Comorbidity Index (CCI) [13]. Both groups were compared in terms of preoperative demographic variables, Dorr classification [14], length of hospital stay, preoperative Harris hip score (HHS) [11], WOMAC index [9], and SF-36 score [10]. Surgical-related parameters were also evaluated including femoral head diameter, femoral stem offset, bearing surface, operative time, and estimated blood loss. The estimated blood loss was calculated according to Nadler’s blood volume formula [15] and Gross’ blood loss formula [16]. All patients were available for follow-up examination at 3 months, 6 months, 1 year, and 2 years postoperatively. All the data were obtained by one observer (K.S.) who was not part of the surgical team. Each postoperative visit included clinical and radiological examination with AP and frog leg lateral views of the pelvis (Table 1).

**Table 1:** Time schedule and outcome measurements pre-operatively and at 3, 6, 12 and 24 months postoperatively

Outcome measure	Specification	Score	Assessment times				
			Pre-op	3 months	6 months	1 year	2 years
*Patient-reported outcome measures*							
WOMAC	General joint pain	0-96	**√**	**√**	**√**	**√**	**√**
SF-36	General health status	0-100	**√**	**√**	**√**	**√**	**√**
*Hip Joint-specific measures*							
Harris Hip score	Joint specific score	0-100	**√**	**√**	**√**	**√**	**√**
*Other*							
Complications			**Surgical**	**√**	**√**	**√**	**√**
Self-administered patient satisfaction	Satisfaction					**√**	**√**

#### Patient-reported outcome measures

All eligible patients completed the WOMAC and SF-36 questionnaires as well as the Harris Hip Score. The WOMAC index, validated in osteoarthritis patients, is a health status questionnaire used to assess pain, stiffness, and function with a varying number of questions for each area (five, two, and seventeen, respectively). It consists of 24 questions in total, assessing the extent of functional limitations in performing a range of daily activities. Responses are provided on a 5-point Likert-type scale. The responses are scored 0–4 on an ordinal scale and include none, mild, moderate, severe, and extreme. Scores range from 0 to 96 for the total WOMAC, with higher scores indicating worse pain, stiffness, and function.

Overall mental and physical wellbeing was checked according to the SF-36 score. The test specifically covers eight distinct areas: physical functioning (PF), role limitations due to physical health (RP), bodily pain (BP), general health (GH), vitality (VT), social functioning (SF), role limitations due to emotional health (RE), and mental health (MH) with a score given for each domain from a composite of 36 questions. Scores are stratified from extreme symptoms/poor health) to 100 (no symptoms/perfect health).

The HHS is a clinician-based hip joint-specific measure, covering domains such as pain, function, absence of deformity, and range of motion. Scores from each domain are summed to give a total ranging from 0 to 100, where higher scores indicate better outcome.

#### Reporting complications/patient satisfaction

All intra-operative and post-operative complications were documented. At 1- and 2-year appointments, patient satisfaction [17, 18] was assessed and categorized as overall satisfaction, satisfaction with pain relief, functional improvement to perform daily activities, and satisfaction with ability to perform recreational activities. Patients were classified as very satisfied, somewhat satisfied, somewhat dissatisfied, and dissatisfied. Clinical evaluation included also the presence or absence of thigh pain.

#### Radiographic evaluation

**Fig. 3:**
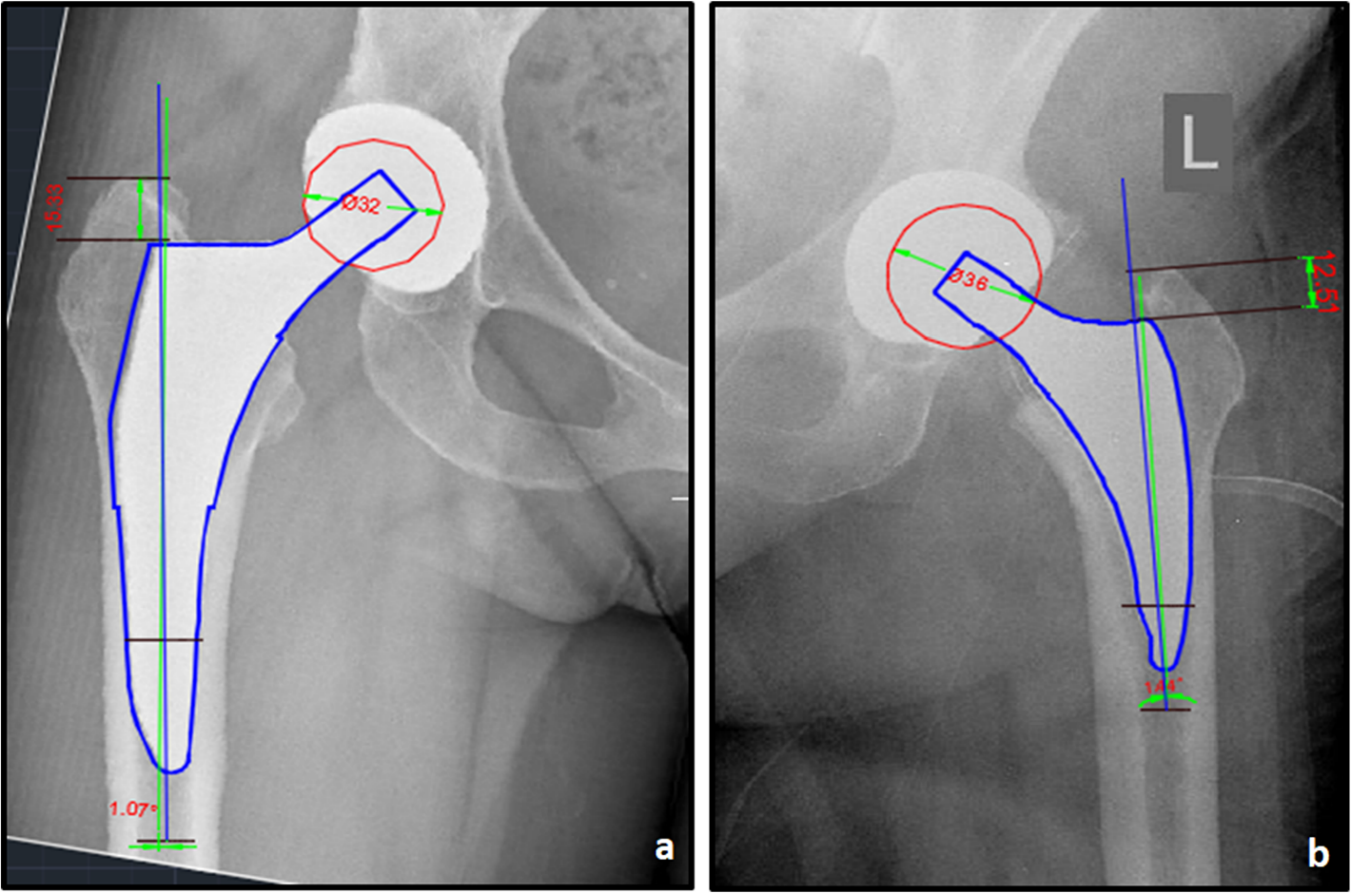
AutoCAD template image on the AP pelvis radiograph showing subsidence calculation and the measurements of stem coronal alignment (angle between the longitudinal axis of the stem—green line, indicated on templates provided by the manufacturer, relative to the axis of the proximal femoral shaft—blue line). Radiographs were calibrated based on the known size of the femoral head. **a** Tri-Lock BPS. **b** Minima S

All antero-posterior pelvis radiographs were obtained in a similar manner with both legs internally rotated 15° and with bony landmarks (teardrop and lesser trochanter) clearly visible. All radiographic measurements were performed with Autocad Mechanical Version 2015 software. To test the reliability of the measurements, all radiographs were reviewed by two independent examiners. Radiographs were calibrated and corrected for any magnification based on the known size of the femoral head. Femoral prosthesis fitting, alignment, and stability were assessed. Varus, valgus, or neutral implant position was checked at the first postoperative radiological assessment by measuring the angulation between the longitudinal axis of the stem relative to the axis of the femoral shaft. Stem alignment was considered normal if its deviation from the axis of the femoral shaft was 5° or less. Varus or valgus inclination of the stem higher than 5° was defined to be malpositioned [19–21]. Stem subsidence was evaluated by measuring the distance between the tip of the greater trochanter and the shoulder of the prosthesis (Fig. 3). In both groups, all follow-up radiographs were examined for signs of bony ingrowth or signs of loosening and were classified as osseointegrated, fibrous stable, or unstable [22]. Indicators of instability were also assessed. A progressive axial subsidence of >3 mm, a varus or valgus shift of > 3°, and the detection of a complete radiolucent line surrounding the surface on both the anteroposterior and the lateral radiographs were considered signs of possible loosening [19–21, 23, 24]. Pedestal formation, hypertrophies, atrophies, seam formations and spot welds, and sclerotic lines in the form of a neocortex as well as periarticular ossifications according to Brooker’s classification [25] were also recorded.

### Statistical analysis

For sample calculation, the minimal clinically important differences (MCID) have been previously reported at 25 points for the WOMAC score, 20 points for the HHS, and 12% difference for the SF-36 score. In order to achieve an 80% power or better, the effect size is expected to lie between 0.25 and 0.6, suggesting a sample size of 45 patients in each group [26].

All quantitative variables were initially tested for normality with the Shapiro-Wilk test, and subsequently, their values were grouped using the two subtypes, Tri-Lock BPS, and Minima S and were compared. The comparison was performed using Student’s *t* test for parametric variables, or Wilcoxon’s rank-sum test for non-parametric variables. A set of baselines versus consecutive follow-up comparisons were executed with Student’s paired *t* test/Wilcoxon’s signed-rank test, depending again on the normality of the variables. All qualitative variables were checked for a potential association with the subtype, Tri-Lock BPS or Minima S, using Pearson’s chi-squared test of independence. Finally, in order to determine reliability of two independent measurements by corresponding observers, intraclass correlation coefficient I was calculated. Statistical tests were two-sided and statistical significance was taken when *p*<0.05. Statistical analysis was performed with the R language and the RStudio IDE, both of which are open-source products.

## Results

Preoperative baseline characteristics of both groups were similar, and no significant differences were recorded in demographic or surgical-related parameters (Table 2). In the Tri-Lock BPS stem group (group A), there were 16 men and 29 women with a mean age of 63.89±8.56 years whereas in the Minima S stem group (group B), there were 23 men and 22 women with a mean age of 63.49±8.16 years (*p*<0.805). The predominant diagnosis in both groups was primary osteoarthritis (68.89% and 66.67%, respectively). The morphology of the proximal femur according to the Dorr classification was not significantly different between the 2 groups.

**Table 2:** Baseline characteristics of the two stem groups

Parameters	Group A	Group B	***P*** value
**No. of patients (hips)**	45(45)	45(45)	-
**Gender, no. (%)**			0.202 ^**c**^
Male	16 (35.56)	23 (51.11)	
Female	29 (64.44)	22 (48.89)	
**Age (years)** ^d^	63.89 ±8.56	63.49±8.16	0.805 ^**b**^
**Weight (kg)** ^d^	80.29±14.06	81.28±14.83	0.942 ^**b**^
**Height (m)** ^d^	1.68±0.08	1.69±0.1	0.764 ^**a**^
**BMI (kg/m**^**2**^**)** ^d^	28.45±4.95	28.52±4.31	0.869 ^**b**^
**CCI** ^d^	2.38±1.21	2.2±1.16	0.541 ^**b**^
**Femoral canal (Dorr type), No. (%)**			0.672 ^**c**^
Type A	12 (26.67)	14 (31.11)	
Type B	28 (62.22)	24 (53.33)	
Type C	5 (11.11)	7 (15.56)	
**Principal diagnosis, no. (%)**			0.519 ^**c**^
Osteonecrosis	2 (4.44)	0 (0)	
DDH	8 (17.78)	10 (22.22)	
Osteoarthritis	31 (68.89)	30 (66.67)	
Traumatic arthritis	4 (8.88)	4 (8.88)	
Rheumatoid arthritis	0 (0)	1 (2.22)	
**Side, no. (%)**			0.397 ^**c**^
Right	22 (48.89)	27 (60)	
Left	23 (51.11)	18 (40)	
**Length of hospital stay (days)** ^d^ **[min., max.]**	4.47 (0.99) [2, 7]	4.4 (0.75) [3, 6]	0.719 ^**b**^
**Estimated blood loss (ml)** ^d^ **[min., max.]**	1037.58 (156.85) [613.14, 1245.39]	994.01 (216.01) [545.86, 1530.17]	0.276 ^**a**^
**Operation time (minutes)** ^d^ **[min., max.]**	64.6 (15.67) [40, 95]	60.18 (12.78) [42, 95]	0.146 ^**a**^
**Femoral stem offset, no. (%)**			0.139 ^**c**^
Standard	20(44.44)	28 (62.22)	
High	25(55.56)	17(37.78)	
**Bearing surface, no. (%)**			0.80 ^**c**^
Ceramic on ceramic	36 (80)	34(75.55)	
Ceramic on polyethylene	9(20)	11(24.45)	
**Femoral head size, no. (%)**			0.673 ^**c**^
32mm	22(48.89)	25(55.56)	
36mm	23(51.11)	20(44.44)	

^a^Student’s *t* test

^b^Wilcoxon’s rank-sum test

^c^Pearson’s chi-squared test

^d^The value is given as the mean and standard deviation

*No.* number, *BMI* body mass index, *CCI* Charlson’s Comorbidity Index, *DDH* developmental hip dysplasia

**Fig. 4:**
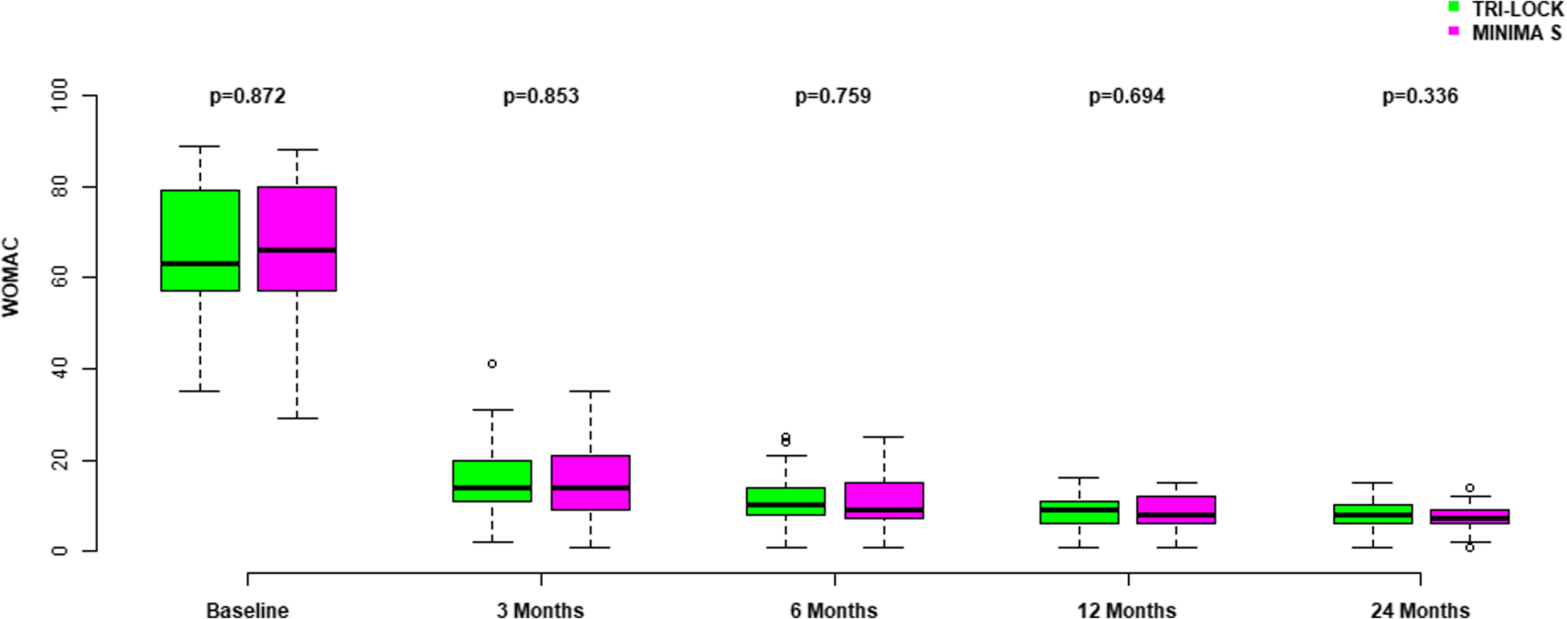
WOMAC scores preoperatively and at 3, 6, 12, and 24 months postoperatively

The baseline WOMAC score was similar between the two groups. At 3 months after THA, WOMAC scores were significantly improved relative to before surgery but no significant differences were recorded between groups. In the consecutive evaluations during follow-up, no significant differences in WOMAC scores were found in the Tri-Lock compared to the Minima S group. From 6 months of follow-up onwards, no significant further improvement was noticed in WOMAC scores (Fig. 4, Table 3).

**Table 3:** Comparison of clinical results in both implant groups at each time point and accompanying *P* values

	Preoperatively			Postoperatively											
				3 m			6 m			1 y			2 y		
	Group A	Group B	***P*** value	Group A	Group B	***P*** value	Group A	Group B	***P*** value	Group A	Group B	***P*** value	Group A	Group B	***P*** value
WOMAC score															
*WP*	14.6 (3.14)	14.11 (3.24)	0.524	2.91 (3.28)	3.13 (3.53)	0.963	1.44 (1.88)	1.38 (2.07)	0.506	0.62 (0.91)	0.6 (0.94)	0.801	0.44 (0.62)	0.36 (0.57)	0.492
*WS*	6 (1.6)	6.16 (1.65)	0.724	1.49 (1.36)	1.44 (1.49)	0.734	0.93 (0.86)	0.84 (0.85)	0.626	0.76 (0.8)	0.71 (0.84)	0.725	0.76 (0.53)	0.62 (0.58)	0.228
*WPh*	45.82 (9.74)	46.09 (11.78)	0.698	11.2 (4.56)	11.07 (5.29)	0.852	8.67 (3.78)	8.8 (4.54)	0.887	7.29 (2.98)	7.07 (3.06)	0.836	7 (2.71)	6.51 (2.74)	0.489
*WT*	66.42 (13.4)	66.36 (15.71)	0.872	15.6 (7.84)	15.64 (9.28)	0.853	11.04 (5.53)	11.02 (6.53)	0.759	8.67 (3.97)	8.38 (4.2)	0.694	8.2 (3.13)	7.49 (3.23)	0.336
SF-36 score															
*PF*	21.56 (13.31)	22.44 (13)	0.98	81.33 (20.04)	77.33 (22.07)	0.305	88.2 2(8.27)	86.67 (8.53)	0.404	88.22 (8.27)	86.67 (8.53)	0.404	88.22 (8.27)	86.67 (8.53)	0.404
*RP*	25.56 (27.95)	18.89 (25.09)	0.234	76.67 (12.39)	77.22 (11.7)	0.84	82.78 (12.86)	83.33 (14.1)	0.787	82.78 (12.86)	84.44 (14.39)	0.497	82.78 (12.86)	84.44 (14.39)	0.497
*BP*	18.9 (13.48)	18.29 (13.89)	0.646	95.17 (7.86)	94.61 (8.29)	0.762	96.67 (6.31)	96.11 (6.96)	0.743	96.67 (6.31)	96.11 (6.96)	0.743	96.67 (6.31)	96.11 (6.96)	0.743
*GH*	60 (17.25)	59.02 (18.46)	0.842	77.56 (13.38)	76.56 (13.73)	0.756	79.33 (11.41)	78.56 (12.04)	0.812	80 (11.13)	79.33 (11.95)	0.873	80 (11.13)	79.33 (11.95)	0.873
*VT*	45.44 (19.65)	45.89 (20.04)	0.675	74.22 (11.58)	73.89 (10.49)	0.869	76.67 (11.18)	76.78 (10.71)	0.706	77 (11.05)	76.11 (10.97)	0.728	77.22 (10.9)	79.56 (10.7)	0.774
*SF*	32.5 (18.73)	32.78 (15.61)	0.752	83.89 (13.75)	82.22 (12.92)	0.509	87.78 (12.92)	86.94 (13.31)	0.792	87.78 (12.92)	87.5 (13.33)	0.969	87.78 (12.92)	87.5 (13.33)	0.969
*RE*	42.99 (39.25)	45.92 (40.39)	0.588	86.68 (17.97)	86.68 (16.5)	0.901	88.16 (16.12)	87.42 (16.33)	0.832	88.16 (16.12)	87.42 (16.33)	0.832	88.16 (16.12)	87.42 (16.33)	0.832
*MH*	56.71 (17.34)	58.04 (20.99)	0.522	77.6 (13.95)	79.56 (13.81)	0.489	79.29 (13.49)	80.27 (13.55)	0.72	79.73 (13.39)	80.44 (13.65)	0.742	79.73 (13.39)	80.44 (13.65)	0.742
HHS															
	46.63 (11.2)	48.8 (12.35)	0.384	91.49 (6.89)	92.34 (8.72)	0.112	95.01 (3.26)	95.79 (3.82)	0.178	95.38 (3)	96.05 (3.53)	0.18	95.38 (2.98)	96.03 (3.56)	0.189

Data presented as mean values (SD)

*SD* standard deviation, *m* month, *y* year(s), *HHS* Harris Hip Score, *WOMAC* Western Ontario and McMaster Universities Arthritis Index, *WP* WOMAC Pain, *WS* WOMAC Stiffness, *WPh* WOMAC Physical Function, *WT* WOMAC Total, *PF* physical functioning, *RP* role limitations due to physical health problems, *BP* bodily pain, *GH* general health, *VT* vitality, *SF* social functioning, *RE* role limitations due to emotional problems, *MH* mental health

**Fig. 5:**
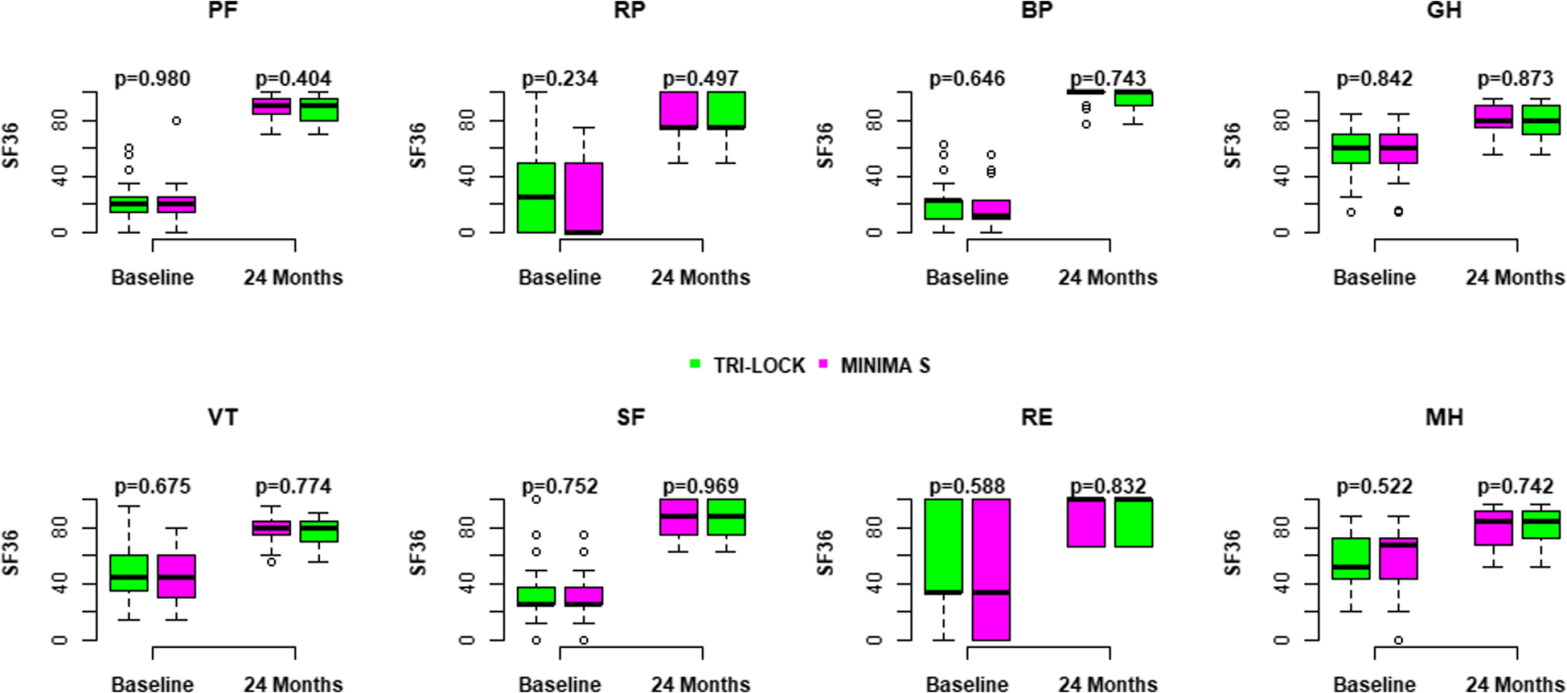
Assessment of each domain of SF-36 score in both groups preoperatively and at 24 months postoperatively

No statistically significant differences in SF-36 scores between the study groups preoperatively and during the follow-up period were found. Both groups demonstrated a similar improvement in each domain of this parameter at 2-year follow-up relative to before surgery. For the majority of these outcomes, statistically significant improvements were found up to 3 months (*p*<0.001 in both groups) compared to the baseline. Since then, the scores remained stable without further improvement (Fig. 5, Table 3).

**Fig. 6:**
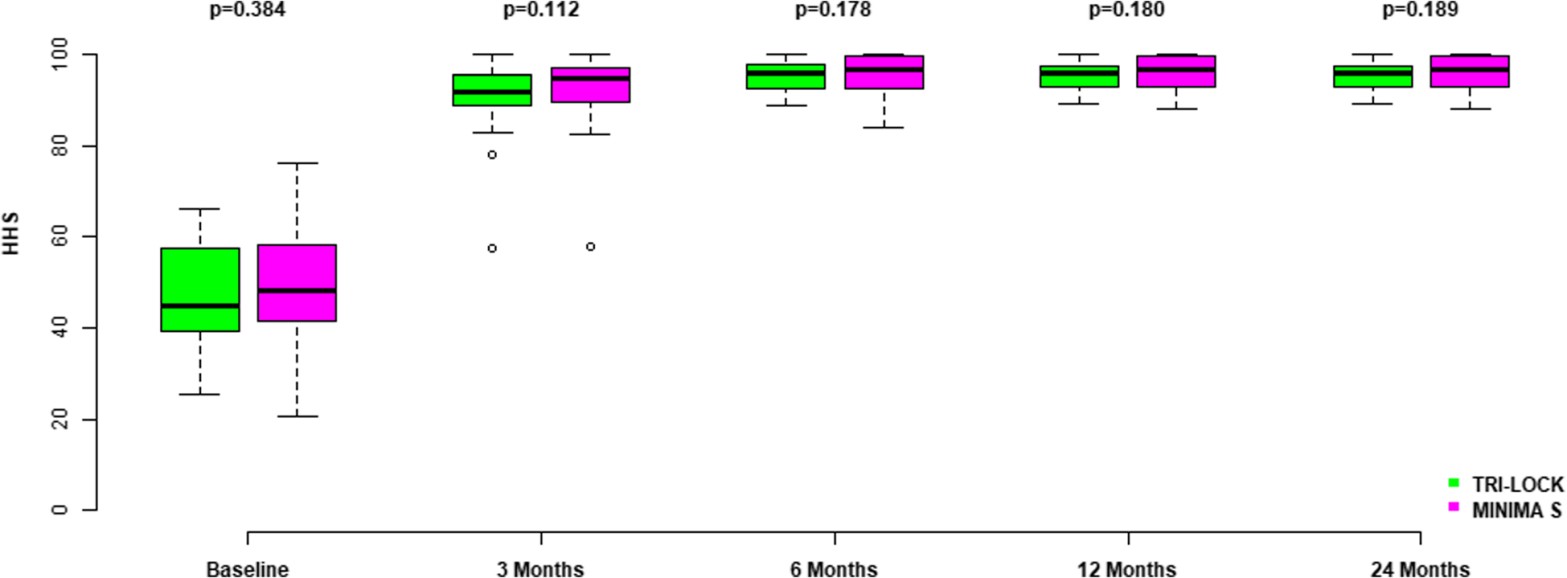
Harris Hip Scores in both groups preoperatively and at 3, 6, 12, and 24 months following implantation

Preoperative Harris hip score was not significantly different between the two groups. Harris hip scores significantly improved in both groups with the most marked improvement achieved within the first 3 months after THA, in comparison to the baseline values (*p*<0.001 in both groups). Patients continued to improve in HHS scores up to the first 6 months after surgery, and no further improvement was noticed during the next follow-up examinations. Moreover, no significant differences in the Harris hip score were found between the two groups at any follow-up appointment (Fig. 6, Table 3).

Data from Patient Satisfaction Questionnaire showed that patients’ overall satisfaction related to the hip surgery and subsequent pain relief was at the highest level of scoring system in both groups. The ratings were also very high and did not differ between the groups regarding satisfaction performing either daily or recreational activities with 88.9% and 86.7% of patients being “very satisfied” or “somewhat satisfied” at 2 years of follow-up, in groups A and B, respectively. In all aspects of satisfaction questionnaire, no further improvement was recorded between the two assessment points (Table 4).

**Table 4:** Level of patient satisfaction postoperatively

	12 months		***P*** value*	24 months		***P*** value*
	Group A	Group B		Group A	Group B	
Q1“Overall how satisfied are you with the results of your hip replacement surgery?” VS, SS/SD, D (n/n, %/%)	45/0 (100/0)	45/0 (100/0)	1	45/0 (100/0)	45/0 (100/0)	1
Q2“How satisfied are you with the results of your hip replacement surgery for relieving your pain?” VS, SS/SD, D (n/n, %/%)	45/0 (100/0)	45/0 (100/0)	1	45/0 (100/0)	45/0 (100/0)	1
Q3 “How satisfied are you with the results of your hip replacement surgery for improving your ability to do home or yard work?” VS, SS/SD, D (n/n, %/%)	44/1 (97.8/2.2)	44/1 (97.8/2.2)	1	44/1 (97.8/2.2)	44/1 (97.8/2.2)	1
Q4 “How satisfied are you with the results of your hip replacement surgery for improving your ability to do recreational activities?” VS, SS/SD, D (n/n, %/%)	38/7 (84.4/15.6)	37/8 (82.2/17.8)	1	40/5 (88.9/11.1)	39/6 (86.7/13.3)	1

*Chi-square test

*Q* question, *n* number, *VS* very satisfied, *SS* somewhat satisfied, *SD* somewhat dissatisfied, *D* dissatisfied

Overall, there was no significant difference in the incidence of complications between the two groups. No intra-operative complications were recorded in either group. There were two early superficial infections in group A (4.4%) and one in group B (2.2%) (*p*=1). These were treated with intravenous antibiotics for 2 weeks and settled unremarkably. Two patients in group A (4.4%) complained for thigh pain, rated 3 and 4 according to VAS (visual analogue scale) pain scale, at 3 months post-surgery that being resolved at the following appointment. One patient (2.2%) in group B reported anterior thigh pain and scored 3 points at VAS at 2 years of follow-up. In this case, femoral stem had 1.39° valgus position, and a grade 2 stress shielding effect was noted but without evidence of other periprosthetic bone alterations up to the last follow-up. There was no significant difference in the incidence of this complication between both groups (*p*=1). In the Tri-Lock BPS group, 6 (13.3%) patients developed heterotopic ossifications (HO); 5 patients at 3 months and 1 patient at 6 months postoperatively, all classified as Brooker class I. In two of them, a further progression to Brooker class II and III HO in respect was found at 1 year and remained stable thereafter. In the Minima S group, 3 (6.7%) patients developed heterotopic ossifications class I; 1 case at 3 months and 2 cases at 6 months, without further progression up to the last follow-up. All cases in both groups were asymptomatic without functional limitations and were treated conservatively. There was no statistically significant difference in the HO incidence between the two groups (*p*=0.521). There were no cases of aseptic loosening in either group within the 2 years of follow-up.

**Fig. 7:**
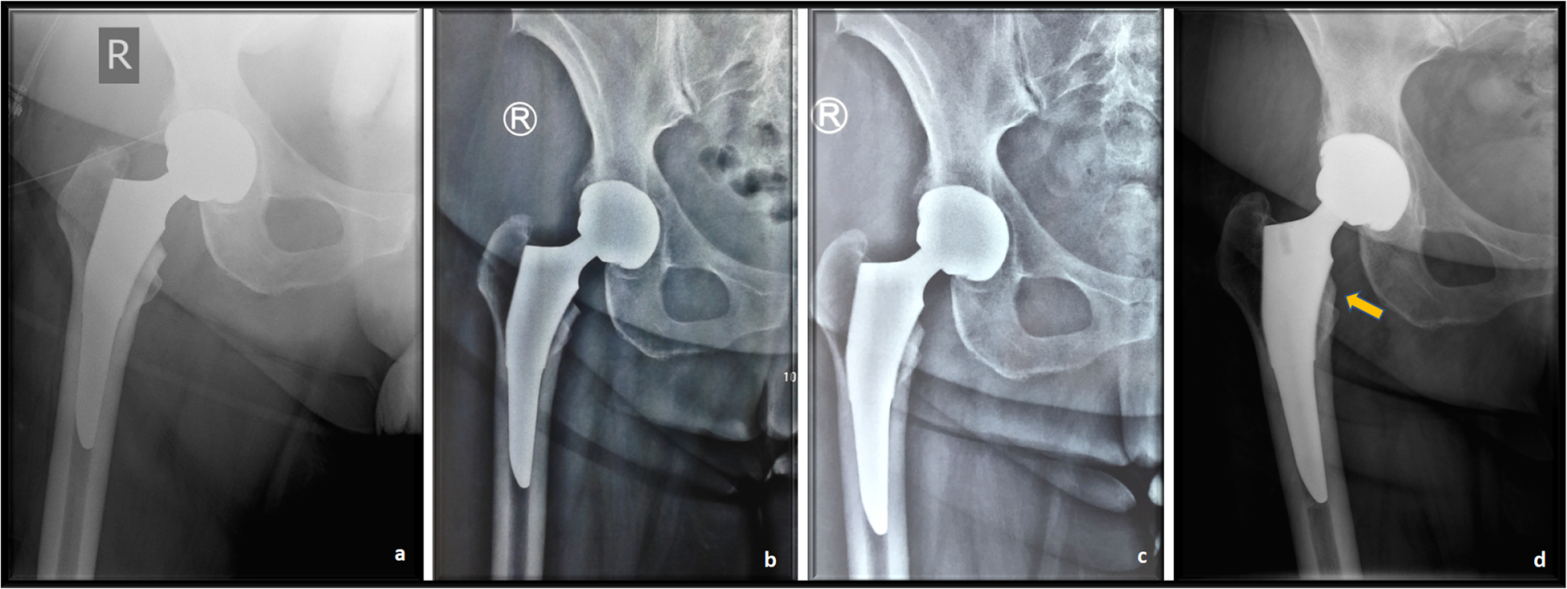
Anteroposterior pelvis radiographs of a 59-year-old woman who underwent a right total hip arthroplasty with the Tri-Lock BPS. **a** First day, **b** 6 months, **c** 1-year, and **d** 2 years postoperatively. Grade 1 stress shielding effect was noted 2 years after implantation

**Fig. 8:**
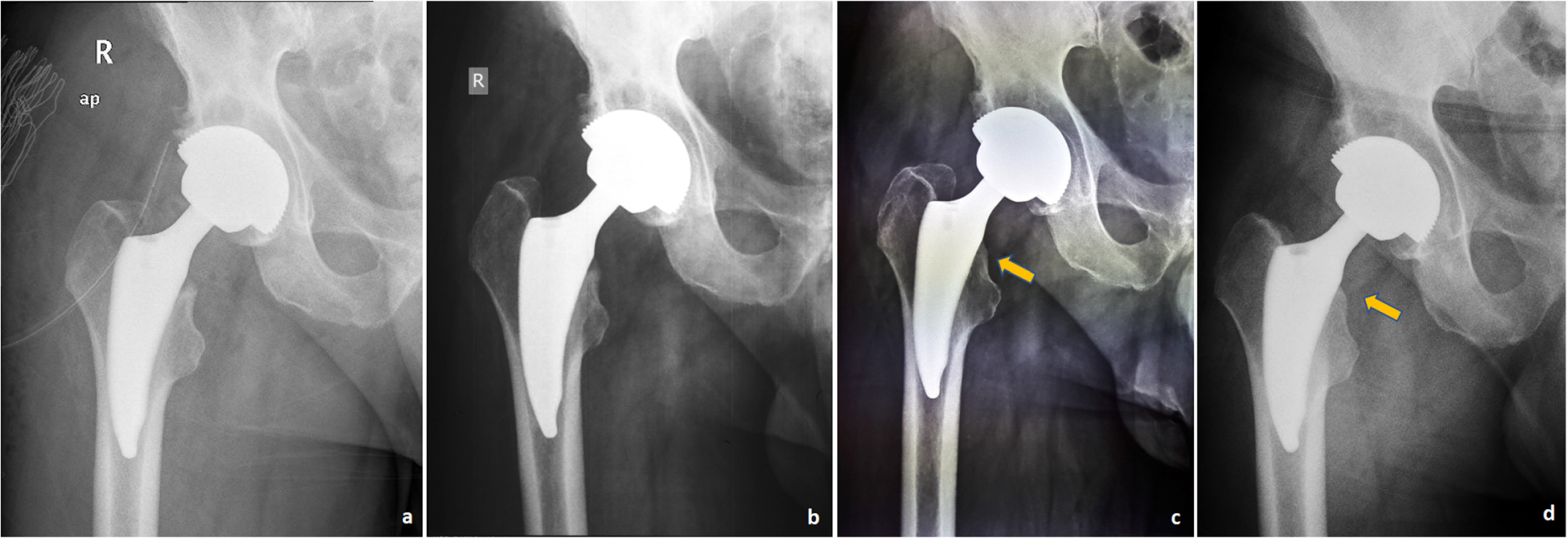
Anteroposterior pelvis radiographs of a 61-year-old man who underwent a right total hip arthroplasty with the Minima S stem. **a** First day, **b** 6 months, **c** 1 year, and **d** 2 years postoperatively. Grade 1 stress shielding effect was noted postoperatively at 1 and 2 years after implantation

Interobserver reliability was assessed and found to be almost perfect for the quantitative radiographic measurements (ICC=0.99). The alignment of the femoral component, the subsidence of the femoral component, the presence of radiolucent lines, and stress shielding-induced bone alterations were assessed during the follow-up period. In group A, the stem was positioned slightly varus (mean angle 0.98°±0.66) in 23 (51.1%) patients and slightly valgus (mean angle, 0.97°±0.5) in 6 (13.3%) patients. In the remaining 16 (35.6%) patients, the Tri-Lock BPS stem was implanted in the exact neutral position. In group B, the stem was placed at slight valgus or varus in 27 (60%) and 18 (40%) patients, respectively, with a mean valgus angle of 1.12° (±0.56) and a mean varus angle of 1.16° (±0.94). An exact superimposable position of the anatomical femoral axis with the alignment axis of the implant was not noticed in any Minima S implanted cases. In terms of femoral component alignment, statistically significant differences were noted between the study groups (*p*<0.001). Nevertheless, no case of stem malposition was recorded in neither group. The relative changes in coronal alignment between the initial postoperative radiographs and the last follow-up radiographs were minimal in both groups. An angle difference of 0.29° (±0.43) and 0.24° (±0.23) was recorded in group A and group B, respectively (*p*=0.174). At the last follow up, a mean subsidence value of 0.87 (±0.56) mm and 0.80 (±0.69) mm was observed compared to the baseline measurements, in the groups A and B, respectively. No statistically significant difference in this parameter was noted between study groups (*p*=0.305). Neither group of patients presented stem subsidence greater than 3 mm. On the contrary, a statistically significant difference between the two groups was recorded in terms of the incidence of stress shielding phenomenon (*p*=0.015). At the last follow-up, in group A, analysis of the calcar region showed that 21 (46.67%) and 5 (11.11%) patients had grade 1 and grade 2 stress shielding effect, respectively. In group B, 12 (26.67%) patients demonstrated slight rounding of the proximal-medial edge of the osteotomized femoral neck, related to grade 1 stress shielding effect and only one patient had grade 2. No hip in either group exhibited grade 3 or higher stress shielding. The incidence of stress shielding phenomenon showed no statistically significant correlation with the observed deviations of coronal stem alignment (*p*=0.914, in group A and *p*= 0.094, in group B). Osseointegration was seen in all the femoral and acetabular components in both groups. No radiographic evidence of periprosthetic osteolytic lesions was detected and no patient required revision surgery for aseptic loosening throughout the entire follow-up period. No radiolucent lines were detected around the surface of any acetabular or femoral components on the AP or lateral radiographs. We recorded two cases (4.4%) of pedestal formation and one case (2.2%) of cortical hypertrophy located in Gruen zone 5, in the Tri-Lock BPS implanted group. There was no correlation between the incidence of periprosthetic bone alterations and stem alignment deviations (*p*=0.558) or the occurrence of thigh pain (*p*=0.818). No similar radiographic alterations were detected in the Minima S implanted group. Representative cases are illustrated in Figs. 7 and 8.

## Discussion

To our knowledge, this is the first study that directly compares different design short femoral stems, classified to the same main category as type 4 according to Khanuja et al. [5], in a randomized fashion using validated clinical measurement tools. More specifically, we decided to assess the clinical and radiological outcomes after the implantation of the newly introduced short stem, Minima S, for which clinical performance data are sparse in the literature [27] with an older generation short stem, the Tri-Lock BPS, with an established record of performance in short to midterm follow-up [28–30]. We have also summarized the short-term implant related complications.

Both patient groups demonstrated significant improvement in terms of functional scoring (HHS, WOMAC, and SF-36) up to 6 months of follow-up compared to the baseline measurements, but no further improvement was noticed at the consecutive follow-up examinations up to 2 years of follow up. HHS, WOMAC, and SF-36 scores reached and remained throughout the remaining follow-up in a plateau state of high values. This observation is consistent with previous studies reporting on the ceiling effect presented by several PROMs (patient-reported outcome measurements) [31, 32]. However, in this study, we did not find a significant difference in these outcomes between the two study groups at any time point. Therefore, no evidence of important clinical difference in the short-term outcome could be provided from the comparison of these two short femoral stems.

According to the review study reported by Khanuja et al. [5], in 3 reports [33–35] involving 294 hips implanted with type 4 short femoral stems, the mean Harris hip score was 93 points. According to a recent systematic review [3] focused on short metaphyseal loading cementless stems, types III or IV by Khanuja et al. [5], the mean Harris Hip Score and WOMAC score improved from 46 (0 to 100) to 92 (39 to 100) and from 54 (2 to 95) to 22 (0 to 98), respectively. In the recent study of Ulivi et al. [28], reported on Tri-Lock BPS femoral stem, HHS increased from a mean of 27.29 (±4.6) preoperatively up to 97.28 (±9.0) at 5 years in a cohort of 163 consecutive patients. Drosos et al. [27] published favorable short-term clinical results after the implantation of Minima S stem in 61 patients, with an improvement in HHS from 58.7 preoperatively to 95.1 postoperatively at a mean follow-up of 2.8 years. These clinical results are consistent with the clinical scores noted in the present study. Specifically, we recorded an improvement in the mean HHS from 46.63 (±11.2) and 48.8 (±12.35) preoperatively to 95.38 (±2.98) and 96.03 (±3.56) at 2 years, in Tri-Lock BPS and Minima S groups, respectively. Similarly, WOMAC scores improved from 66.42 (±13.4) and 66.36 (±15.71) to 8.2± (3.13) and 7.49 (±3.23) at the last follow-up. These results are considered undoubtedly in line with the expectations of the modern THA.

Regarding the incidence of complications, there were no significant differences between the two study groups. Two patients in group A complained of thigh pain at 3 months that resolved up to the next appointment. Only 1 patient (2.2%) in group B had persistent thigh pain at the last follow-up. In our study, the low incidence of thigh pain is in accordance with the previous systematic review of Lidder et al. [3] on short metaphyseal femoral stems, reporting an incidence of 0.19% (range 0 to 6.9%). On the contrary, Amendola et al. [29], in 261 hips implanted with the Tri-Lock BPS, showed high incidence (22.6% of patients) of thigh pain although the HHS improved from 47 to 88. To our knowledge, there are no similar studies on metaphyseal fitting short stems, reporting such high rates in the incidence of this complication. In agreement with previous studies [3, 5, 28], that have reported a low incidence of periprosthetic fractures with these types of stems, in this study, no cases of periprosthetic fractures were observed in neither group.

In this study, regarding coronal alignment, no case of stem malposition was recorded in neither group. Nevertheless, statistically significant differences were noted between the study groups (*p*<.001) in this radiological parameter. In group A, the stem was placed in slight varus (mean angle of 0.98°) and valgus (mean angle of 0.97°) position in 51.1% and 13.3% of patients, respectively. In 35.6% of patients, Tri-Lock BPS stem was implanted in the exact neutral position. The Minima S stem was implanted at slight varus (mean angle of 1.16°) and valgus (mean angle of 1.12°) in 60% and 40% of patients, respectively. In group B, neither stem was placed in the exact neutral position. We believe that the shorter design of the Minima S implant and its limited extension to the proximal diaphysis compared to the Tri-Lock BPS stem interprets the discrepancies observed in the coronal alignment of this implant. However, the minimal reported differences in terms of alignment for both femoral stems had no effect on implants’ survival and patient-reported outcomes. Generally, a particular concern has been raised with short stem implants regarding the higher rate of coronal malalignment compared to the conventional standard length femoral implants [5]. The fact is that short stems lack appropriate distal extension into the diaphysis and therefore prevent the surgeon from guiding them correctly during implantation [36]. According to the systematic review conducted by Lidder et al. [3], regarding the alignment deviations of the short components from the neutral axis, there were no significant differences in survivorship and clinical outcomes between the varus aligned components compared to those in a neutral position. Nevertheless, looking ahead and taking into consideration the short to midterm results of short femoral components, further investigation into the prevalence and long-term consequences of this notable parameter is required.

Because the presence or absence of signs of instability was of particular concern in this study, we assessed the subsidence as well as the relative changes in stem alignment between the initial postoperative and the last follow-up radiographs. No statistically significant differences in these parameters were noted between the study groups. In both groups, the observed values of subsidence and angle difference of coronal alignment are consistent with previous results [27, 28, 30] and below the previously stated thresholds for loosening [37, 38]. Furthermore, all femoral components were considered osseointegrated in both groups, without evidence of periprosthetic osteolytic lesions at the last follow-up.

In the present study, there was a statistically significant difference between the two groups in terms of the incidence of stress shielding phenomenon (*p*=0.015). At the last follow-up, the analysis of the calcar region showed that 57.8% patients in group A and 28.9% in group B had grades 1 or grade 2 stress shielding. No hip in either group exhibited grade 3 or higher stress shielding. These results are in agreement with previous reports focused on the radiological outcomes of these particular femoral stems. Drosos et al. [27] noted proximal femoral stress shielding grade 1 in all enrolled patients undergone THA with the Minima S femoral stem. Yu et al. [39] published on 55 hips implanted with Tri-Lock BPS and found grade 1 stress shielding effect in all consecutive patients. Amendola et al. [29] reported similar findings in a series of 212 Tri-Lock BPS implanted hips. Furthermore, the results of the present study are in line with the conclusions of our previous experimental investigation [40], focused on the biomechanical behavior of the Tri-Lock BPS and Minima S femoral stem using digital image correlation. We demonstrated that stress shielding effect cannot be avoided since a proximal unloading of the femur was noted after the implantation of both short stem designs. However, the specific dissimilarities in strain distributions induced after the implantation of each prosthesis due to their design specific variations did not induce a clinical visible impact on the radiological parameters investigated in the current comparative clinical study.

We are aware of several limitations in this study. At first, we are not able to draw conclusions about the long-term performance of these femoral stems due to the short-term follow-up period. Nevertheless, in accordance with previous studies [41–44], key parameters to achieve a long-term stable fixation are considered to be the initial fitting and the prevention of early progressive migration of the prosthesis. Furthermore, the fact that many innovative hip implants have been easily adopted in clinical practice without sound premarketing testing supports the need for prospective high-quality studies enabling the detection of early failures or at least the evaluation of their short-term clinical performance. Additionally, we conducted a qualitative assessment of bone remodeling and stress shielding effect from plain radiographs that lack the quantitative accuracy of radiostereometric analysis (RSA) [45, 46] and dual-energy X-ray analysis (DEXA) [47]. However, we used an established grading system that enables the evaluation of bone remodeling on plain radiographs [48]. We also acknowledge that the determination of subsidence and coronal alignment of the implanted stems could be biased by the variability of radiographic quality and observer interpretations. In this study, interobserver reliability was assessed and found to be almost perfect for all radiographic measurements. Another weakness of this study is that all operations were performed by a single experienced surgeon in a single center, and thus, the reported results could potentially reflect one surgeon’s experience. For this reason, further multicenter studies are needed for more consistent results.

## Conclusions

In conclusion, both type 4, different design short femoral stems demonstrated excellent clinical performance and very low incidence of complications up to 2 years of follow-up without any evidence of differences in patient-reported outcomes. In both groups, stable fixation and radiographic osseointegration were achieved, with no cases of aseptic loosening, progressive radiolucent lines, or periprosthetic osteolysis. Although our results are in accordance with previous reports, concerns have been raised regarding the incidence of stress shielding phenomenon and mild discrepancies in coronal stem alignment during implantation. Further multicenter studies with larger cohorts and longer follow-up are needed to evaluate the clinical impact of these concerns if any and also the long-term performance and survivorship of short femoral stems.

## Data Availability

The datasets used and analyzed during the current clinical study are available from the corresponding author on reasonable request.

## Abbreviations

THA: Total hip arthroplasty
WOMAC: Western Ontario and McMaster Universities Osteoarthritis Index
SF-36: 36-Item Short Form Health Survey
HHS: Harris hip score
Tri-Lock BPS: Tri-Lock Bone Preservation Stem
ISRCTN: International Standard Randomized Controlled Trial Number
BMI: Body mass index
CCI: Charlson’s Comorbidity Index
PF: Physical functioning
RP: Role limitations due to physical health
BP: Bodily pain
GH: General health
VT: Vitality
SF: Social functioning
RE: Role limitations due to emotional health
MH: Mental health
MCID: Minimal clinically important differences
VAS: Visual analogue scale
HO: Heterotopic ossification
PROMs: Patient Reported Outcome Measurements
RSA: Radiostereometric analysis
DEXA: Dual-energy x-ray analysis

## References

[1] Huo SC, Wang F, Dong LJ, et al. Short-stem prostheses in primary total hip arthroplasty: a meta-analysis of randomized controlled trials. Medicine (Baltimore). 2016;95(43):e5215. 10.1097/MD.0000000000005215. 10.1097/MD.0000000000005215 PMC5089112 27787383

[2] Castelli CC, Rizzi L (2014). Short stems in total hip replacement: current status and future. Hip Int.

[3] Lidder S, Epstein DJ, Scott G (2019). A systematic review of short metaphyseal loading cementless stems in hip arthroplasty. Bone Joint J.

[4] Schmidutz F, Grote S, Pietschmann M, Weber P, Mazoochian F, Fottner A, Jansson V (2012). Sports activity after short-stem hip arthroplasty. Am J Sports Med..

[5] Khanuja HS, Banerjee S, Jain D, Pivec R, Mont MA (2014). Short bone-conserving stems in cementless hip arthroplasty. J Bone Joint Surg Am..

[6] Feyen H, Shimmin AJ (2014). Is the length of the femoral component important in primary total hip replacement?. Bone Joint J..

[7] Falez F, Casella F, Papalia M (2015). Current concepts, classification, and results in short stem hip arthroplasty. Orthopedics..

[8] Gómez-García F, Fernández-Fairen M, Espinosa-Mendoza RL (2016). A proposal for the study of cementless short-stem hip prostheses. Una propuesta para el estudio de prótesis de cadera de vástago corto. Acta Ortop Mex..

[9] Bellamy N, Buchanan WW, Goldsmith CH, Campbell J, Stitt LW (1988). Validation study of WOMAC: a health status instrument for measuring clinically important patient relevant outcomes to antirheumatic drug therapy in patients with osteoarthritis of the hip or knee. J Rheumatol..

[10] Brazier JE, Harper R, Jones NM, O'Cathain A, Thomas KJ, Usherwood T, Westlake L (1992). Validating the SF-36 health survey questionnaire: new outcome measure for primary care. BMJ..

[11] Harris WH (1969). Traumatic arthritis of the hip after dislocation and acetabular fractures: treatment by mold arthroplasty. An end-result study using a new method of result evaluation. J Bone Joint Surg Am..

[12] Tatani I, Panagopoulos A, Diamantakos I, Sakellaropoulos G, Pantelakis S, Megas P (2019). Comparison of two metaphyseal-fitting (short) femoral stems in primary total hip arthroplasty: study protocol for a prospective randomized clinical trial with additional biomechanical testing and finite element analysis. Trials..

[13] Charlson ME, Pompei P, Ales KL, MacKenzie CR (1987). A new method of classifying prognostic comorbidity in longitudinal studies: development and validation. J Chronic Dis..

[14] Dorr LD, Faugere MC, Mackel AM, Gruen TA, Bognar B, Malluche HH (1993). Structural and cellular assessment of bone quality of proximal femur. Bone..

[15] Nadler SB, Hidalgo JHBT (1962). Prediction of blood volume in normal human adults. Surgery.

[16] Gross JB (1983). Estimating allowable blood loss: corrected for dilution. Anesthesiology..

[17] Mahomed N, Gandhi R, Daltroy L, Katz JN (2011). The self-administered patient satisfaction scale for primary hip and knee arthroplasty. Arthritis..

[18] Mahomed NN, Liang MH, Cook EF, Daltroy LH, Fortin PR, Fossel AH, Katz JN (2002). The importance of patient expectations in predicting functional outcomes after total joint arthroplasty. J Rheumatol..

[19] Kim YH, Kim JS, Joo JH, Park JW (2012). A prospective short-term outcome study of a short metaphyseal fitting total hip arthroplasty. J Arthroplasty..

[20] Kim YH, Kim JS, Oh SH, Kim JM (2003). Comparison of porous-coated titanium femoral stems with and without hydroxyapatite coating. J Bone Joint Surg Am..

[21] Min BW, Song KS, Bae KC, Cho CH, Kang CH, Kim SY (2008). The effect of stem alignment on results of total hip arthroplasty with a cementless tapered-wedge femoral component. J Arthroplasty.

[22] Engh CA, Massin P, Suthers KE (1990). Roentgenographic assessment of the biologic fixation of porous-surfaced femoral components. Clin Orthop Relat Res..

[23] Barreca S, Ciriaco L, Ferlazzo M, Rosa MA (2015). Mechanical and biological results of short-stem hip implants: consideration on a series of 74 cases. Musculoskelet Surg..

[24] Engh CA, Culpepper WJ, Engh CA (1997). Long-term results of use of the anatomic medullary locking prosthesis in total hip arthroplasty. J Bone Joint Surg Am..

[25] Brooker AF, Bowerman JW, Robinson RA, Riley LH (1973). Ectopic ossification following total hip replacement. Incidence and a method of classification. J Bone Joint Surg Am..

[26] Faul F, Erdfelder E, Lang A-G, Buchner A (2007). G*Power 3: A flexible statistical power analysis program for the social, behavioral, and biomedical sciences. Behavior Research Methods.

[27] Drosos GI, Tottas S, Kougioumtzis I, Tilkeridis K, Chatzipapas C, Ververidis A (2020). Total hip replacement using MINIMA® short stem: a short-term follow-up study. World J Orthop..

[28] Ulivi M, Orlandini LC, Meroni V, Lombardo MDM, Peretti GM (2018). Clinical performance, patient reported outcome, and radiological results of a short, tapered, porous, proximally coated cementless femoral stem: results up to seven years of follow-up. J Arthroplasty..

[29] Amendola RL, Goetz DD, Liu SS, Callaghan JJ (2017). Two- to 4-year followup of a short stem THA construct: excellent fixation, thigh pain a concern. Clin Orthop Relat Res..

[30] Albers A, Aoude AA, Zukor DJ, Huk OL, Antoniou J, Tanzer M (2016). Favorable results of a short, tapered, highly porous, proximally coated cementless femoral stem at a minimum 4-year follow-up. J Arthroplasty..

[31] Wang L, Zhang Z, McArdle JJ, Salthouse TA (2009). Investigating ceiling effects in longitudinal data analysis. Multivariate Behav Res..

[32] van Oldenrijk J, Scholtes VAB, van Beers LWAH, Geerdink CH, Niers BBAM, Runne W, Bhandari M, Poolman RW, CUSTOM trial research collaborative (2017). Better early functional outcome after short stem total hip arthroplasty? A prospective blinded randomised controlled multicentre trial comparing the Collum Femoris Preserving stem with a Zweymuller straight cementless stem total hip replacement for the treatment of primary osteoarthritis of the hip. BMJ Open..

[33] Molli RG, Lombardi AV, Berend KR, Adams JB, Sneller MA (2012). A short tapered stem reduces intraoperative complications in primary total hip arthroplasty. Clin Orthop Relat Res..

[34] Stulberg SD, Dolan M (2008). The short stem: a thinking man’s alternative to surface replacement. Orthopedics..

[35] Patel RM, Lo WM, Cayo MA, Dolan MM, Stulberg SD (2013). Stable, dependable fixation of short-stem femoral implants at 5 years. Orthopedics..

[36] Stulberg SD, Patel RM (2013). The short stem: promises and pitfalls. Bone Joint J.

[37] Kim YH, Kim VE (1993). Early migration of uncemented porous coated anatomic femoral component related to aseptic loosening. Clin Orthop Relat Res..

[38] Engh CA, Massin P (1989). Cementless total hip arthroplasty using the anatomic medullary locking stem. Results using a survivorship analysis. Clin Orthop Relat Res.

[39] Yu H, Liu H, Jia M, Hu Y, Zhang Y (2016). A comparison of a short versus a conventional femoral cementless stem in total hip arthroplasty in patients 70 years and older. J Orthop Surg Res..

[40] Tatani I, Megas P, Panagopoulos A, Diamantakos I, Nanopoulos P, Pantelakis S (2020). Comparative analysis of the biomechanical behavior of two different design metaphyseal-fitting short stems using digital image correlation. Biomed Eng Online..

[41] Engh CA, Bobyn JD, Glassman AH (1987). Porous-coated hip replacement. The factors governing bone ingrowth, stress shielding, and clinical results. J Bone Joint Surg Br..

[42] Itami Y, Akamatsu N, Tomita Y, Nagai M, Nakajima I (1983). A clinical study of the results of cementless total hip replacement. Arch Orthop Trauma Surg..

[43] Kim YH, Kim VE (1993). Uncemented porous-coated anatomic total hip replacement. Results at six years in a consecutive series. J Bone Joint Surg Br..

[44] Khalily C, Whiteside LA (1998). Predictive value of early radiographic findings in cementless total hip arthroplasty femoral components: an 8- to 12-year follow-up. J Arthroplasty..

[45] Kärrholm J. Roentgen stereophotogrammetry. Review of orthopedic applications. Acta Orthop Scand. 1989;60(4):491-503. 10.3109/17453678909149328. 10.3109/17453678909149328 2683567

[46] Malchau H, Kärrholm J, Wang YX, Herberts P (1995). Accuracy of migration analysis in hip arthroplasty. Digitized and conventional radiography, compared to radiostereometry in 51 patients. Acta Orthop Scand..

[47] Cohen B, Rushton N (1995). Accuracy of DEXA measurement of bone mineral density after total hip arthroplasty. J Bone Joint Surg Br..

[48] Engh CA, McAuley JP, Sychterz CJ, Sacco ME, Engh CA (2000). The accuracy and reproducibility of radiographic assessment of stress-shielding. A postmortem analysis. J Bone Joint Surg Am..

